# Biochemical and genomic identification of novel biomarkers in progressive sarcoidosis: HBEGF, eNAMPT, and ANG-2

**DOI:** 10.3389/fmed.2022.1012827

**Published:** 2022-10-25

**Authors:** Nancy G. Casanova, Vivian Reyes-Hernon, Taylor Gregory, Belinda Sun, Tadeo Bermudez, Matthew K. Hufford, Radu C. Oita, Sara M. Camp, Gabriela Hernandez-Molina, Jorge Rojas Serrano, Xiaoguang Sun, Jocelyn Fimbres, Mehdi Mirsaeidi, Saad Sammani, Christian Bime, Joe G. N. Garcia

**Affiliations:** ^1^Department of Medicine, University of Arizona Health Sciences, Tucson, AZ, United States; ^2^Department of Pathology, University of Arizona Health Sciences, Tucson, AZ, United States; ^3^Instituto Nacional de Ciencias Médicas y Nutrición Salvador Zubirán, México City, Mexico; ^4^Instituto Nacional de Enfermedades Respiratorias, México City, Mexico; ^5^Department of Medicine, College of Medicine, University of Florida, Jacksonville, FL, United States

**Keywords:** biomarker, sarcoidosis, fibrosis, plasma, gene expression

## Abstract

**Background:**

Progressive pulmonary fibrosis is a serious complication in subjects with sarcoidosis. The absence of reliable, non-invasive biomarkers that detect early progression exacerbates the difficulty in predicting sarcoidosis severity. To potentially address this unmet need, we evaluated a panel of markers for an association with sarcoidosis progression (HBEGF, NAMPT, IL1-RA, IL-6, IL-8, ANG-2). This panel encompasses proteins related to inflammation, vascular injury, cell proliferation, and fibroblast mitogenesis processes.

**Methods:**

Plasma biomarker levels and biomarker protein expression in lung and lymph nodes tissues (immunohistochemical studies) from sarcoidosis subjects with limited disease and progressive (complicated) sarcoidosis were performed. Gene expression of the protein-coding genes included in this panel was analyzed using RNAseq in sarcoidosis granulomatous tissues from lung and lymph nodes.

**Results:**

Except for IL-8, plasma levels of each biomarker—eNAMPT, IL-1RA, IL-6, ANG-2, and HBEGF—were significantly elevated in sarcoidosis subjects compared to controls. In addition, plasma levels of HBEGF were elevated in complicated sarcoidosis, while eNAMPT and ANG-2 were observed to serve as markers of lung fibrosis in a subgroup of complicated sarcoidosis. Genomic studies corroborated *HBEGF and NAMPT* among the top dysregulated genes and identified cytokine-related and fibrotic pathways in lung granulomatous tissues from sarcoidosis.

**Conclusion:**

These findings suggest HBEGF, eNAMPT, and ANG-2 may serve as potential novel indicators of the clinical severity of sarcoidosis disease.

## Introduction

Sarcoidosis is a multisystemic disease with 20% of afflicted patients estimated to develop progressive lung fibrosis (1). Despite attempts at utilizing genomic (2, 3) and genetic modalities (4–6), the development of progressive pulmonary fibrosis, i.e., complicated sarcoidosis, currently evades prediction due to the absence of reliable, non-invasive biomarkers of early progression. In prior sarcoidosis gene expression profiling studies in peripheral blood mononuclear cells (PBMCs) designed to address this important need, we showed dysregulated candidate genes associated with T cell receptor, cytokine-cytokine, and Jak-STAT signaling in complicated progressive phenotypes (2, 3). Similar studies in pulmonary fibrosis, analyzed peripheral blood gene expression, identifying transcripts associated with disease severity (7); including the activation of STAT3 by IL-6 in progressive fibrosis (8). Interrogation of specific up-regulated genes that link to sarcoidosis severity identified the *HBEGF* gene as differentially expressed in sarcoidosis compared to healthy controls and accurately discriminated subjects with limited sarcoidosis from subjects with progressive lung sarcoidosis (2), *HBEGF* encodes HBEGF (heparin-binding epidermal growth factor), a member of the EGF growth factor family expressed in the bronchial epithelium, smooth muscle, CD4 + T cells, and cardiac muscle. HBEGF signaling is a critical contributor to repair and regeneration processes that restore tissue homeostasis following injury, promoting wound healing, angiogenesis, and adipogenesis (9). The finding of increased *HBEGF* expression linked to disease severity suggested a potential utility of HBEGF as a plasma-based sarcoidosis biomarker.

Identical genomic approaches in subjects with ARDS and pulmonary hypertension (10–13) identified extracellular nicotinamide phosphoribosyl transferase (eNAMPT) as a damage-associated molecular pattern protein (DAMP) and master regulator of innate immunity pathways *via* ligation of the Toll-Like receptor 4 (TLR4) and subsequent NF-κB activation (14), processes directly implicated involvement in organ fibrosis (15, 16). Angiopoietin2 (ANG-2) is an endothelial-derived protein that increases junction instability (17) and has been implicated in directly controlling multiple inflammatory-related signals. Elevated ANG-2, along with circulating levels of eNAMPT, Interleukin-6 (IL-6), and Interleukin-1 receptor agonist (IL-1RA) were predictors of 28-day mortality in ARDS subjects (10, 18). In sarcoidosis, a *IL-1a* SNP was significantly overrepresented in sarcoidosis subjects (compared to controls) (19). IL-6 has been postulated as an important proinflammatory cytokine involved in Th1-mediated immunopathobiology of sarcoidosis (20).

Based upon these genomic and epigenomic studies identifying potentially novel markers in sarcoidosis and other inflammatory lung disorders (19–25), the current study selected HBEGF, NAMPT, IL-1RA, IL-6, IL-8, Ang-2 as a panel of plasma biomarkers postulated as potentially useful for identifying subjects with progressive sarcoidosis and lung fibrosis. In addition to measurements of this panel of plasma biomarkers in 127 sarcoidosis subjects and 82 healthy controls, we performed immunohistochemistry (IHC) studies and RNA sequencing to validate the potential contribution for these biomarkers to serve as diagnostic and prognostic biomarkers in subjects with sarcoidosis. These complementary biochemical and genomic approaches indicate that HBEGF, eNAMPT, and ANG-2 may serve as potentially novel indicators of the clinical severity of sarcoidosis.

## Materials and methods

### Cohort samples and demographics

A total of 127 plasma samples from sarcoidosis and 82 healthy controls were analyzed. The clinical characteristics and demographic information are presented in Table 1. Subjects with sarcoidosis were stratified for complicated phenotypes (*n* = 62). Complicated sarcoidosis was assigned to those exhibiting lung involvement with parenchymal lung disease by CT scan or radiographic Scadding stages III and IV; Forced vital capacity (FVC) < 50%; and cardiac or neurological involvement in addition to lung involvement, as previously described (26). We also identified within the complicated group a subgroup of sarcoidosis with pulmonary fibrosis (*n* = 19) and analyzed this subphenotype independently. For those with missing data for chest involvement, we used the ICD-9 and ICD10 codes to define the complicated status. Written informed consent was obtained from all participating subjects with protocols approved by the Institutional Review Board (IRB) at each participating institution. University of Arizona (IRB #1509097312R001), University of Miami (IRB# 20150612). De-identified samples were also obtained from a sarcoidosis cohort from the Instituto Nacional de Ciencias Medicas y Nutricion in Mexico City (Ref.#1711).

**TABLE 1:** Patient characteristics.

Characteristics	Sarcoidosis (127)	Healthy (82)	*P*-value
Age (mean, *SD*)	55.1, 11.9	57.0, 16.7	0.37
Female	65.4	40.2	<0.01
Race (W, AA, NA)	48.8, 31.5, 0	82, 9, 1.2	<0.01
Ethnicity (Hispanic)	11.0	15.8	0.31
Complicated*	48.8	NA	NA
Scadding stage		NA	NA
0	5.5		
I	6.3		
II	15.0		
III	14.2		
IV **	15.0		
Organ involvement		NA	NA
Neurological	9.4		
Eye	58.8		
Cardiac	8.7		
Other (Bone, liver, skin)	15.7		

Age was reported in mean ± standard deviation (sd). Numbers in the rest of the variables are reported as percentages. Race, W, White non-Hispanic; AA, African American; NA, Native American.

*Complicated status was defined according to the following parameters: Lung involvement: Documented parenchymal lung disease by CT scan or radiographic stages III and IV and/or PFT with FVC <50%. Organ involvement: Cardiac or neurological involvement.

**Stage IV pulmonary fibrosis: according to the chest radiographic or CT scan classification (hilar retraction, bullae, cysts, ground-glass opacities, honeycombing, and emphysema).

### Plasma biomarker detection

Plasma was obtained from venous blood collected in EDTA tubes. Following centrifugation (2,500 × g for 15 min), samples were stored at ≤ –70^°^C until biomarker measurement was conducted. 150 μl of plasma was used to quantify HBEGF levels using Quantikine^®^ ELISA Human HBEGF immunoassay (R&D Systems^®^), quantitative sandwich enzyme immunoassay technique. Plasma levels of eNAMPT, IL-6, IL-8, Ang-2, and IL-1RA, were measured in 50 μl of plasma utilizing an electrochemiluminescent multiplex immunoassay predesigned panel from MesoScale (Meso Scale Discovery, MSD^®^) (27–29) (see details in Supplementary materials and methods). Supplementary Table 1 shows the detection limits for each biomarker on every assay.

### Immunohistochemistry analyses

Formalin-fixed, paraffin-embedded lung tissues were obtained from biopsy or autopsy specimens of individuals with sarcoidosis (*n* = 11) and healthy controls (*n* = 5) in compliance with their respective IRB. Lung (*n* = 8) and lymph node (*n* = 8) tissues fixed in 10% formalin underwent H&E and IHC staining for three different biomarkers (HBEGF, ANG-2, and NAMPT) using anti-Human HBEGF antibody (R&D Systems, AF-259-NA), ANG-2 recombinant rabbit mAb (ThermoFisher, JM71-34), and rabbit anti-human NAMPT pAb (Bethyl Laboratories, Montgomery, TX, USA) as previously described (27). Histopathology images, graded by a pathologist blinded to study groups, were selected for quantification analysis of H&E, HBEGF, ANG-2, and NAMPT staining area by Image J software version 1.53 h (30).

### Gene expression

Previously generated RNA sequencing datasets for sarcoidosis granulomatous lung and lymph node tissues and healthy tissue were utilized to assess the transcriptomic profile of targeted genes, GO accession number: GSE157671. Gene expression was assayed using an Illumina HiSeq 2000 HTG EdgeSeq™ Oncology-biomarker panel. The differential expression analysis was performed using the Limma package (31). Transcripts with a fold change (FC) > 2 and a *q*-value < 0.01 were deemed as differentially expressed. Detailed methods and analysis have been previously reported (32).

### Statistical analysis

Numerical variables were analyzed using non-parametric Mann–Whitney test and Kruskal–Wallis test when parametric assumptions were not met. Group comparisons were performed using Spearman correlation. Sensitivity and specificity of the variables were calculated to evaluate the area under the curve (AUC) of the Receiver Operator characteristics (ROC) in each of the plasma markers. Cut-off values were selected using J statistics (Youden index). A significance of 0.05% (*p* < 0.05) was required to consider statistical significance. All analyses were performed with Stata v.17 (StataCorp, TX), and Graphpad Prism v. 8.0 software (San Diego, CA).

## Results

### Patient characteristics

The patient characteristics in the sarcoidosis (*n* = 127) and healthy control groups (*n* = 82) are presented in Table 1. There was no significant difference in age between groups (*p* = 0.37). The sarcoidosis group exhibited a higher proportion of females and African Americans compared to controls (*p* < 0.01). Radiographic or chest CT scan data indicating lung involvement stage was available on 71 of the 127 sarcoidosis subjects, with pulmonary fibrosis reported in 19 (15%) of total sarcoidosis subjects. 62 subjects (49%) of the sarcoidosis cohort were classified as complicated sarcoidosis; documented parenchymal lung disease by CT scan or radiographic Scadding stages III and IV; FVC < 50%; and/or cardiac or neurological involvement. The characteristics of the complicated and uncomplicated groups are presented in Table 2.

**TABLE 2:** Characteristics of the phenotypic groups.

Characteristics	Complicated (62)	Uncomplicated (63)
Age (Mean, *SD*)	57.5, 10.8	54.9, 12
Race (Black, W, NA)	47, 51, 6	35, 57,8
Chest imaging available	69.3	42.9
Stages 0–II	0	38.1
Stages III–IV	56.5	0
Lung fibrosis	30.6	0
**Organ involvement**		
Neurological/cardiac	37.1	0
Other*	46.8	23.8
Absent chest imaging, positive neurological, cardiac or lung involvement reported	30.6	0
Absent chest imaging and no lung, cardiac or neurological involvement reported	0	58.7

Age is reported in mean ± standard deviation (SD). Numbers in the rest of the variables are reported as percentages. Race, W, White non-Hispanic; AA, African American; NA, Native American.

*Other organ involvement: eye, bone, joints, liver, kidney, or skin.

### Circulating biomarkers in sarcoidosis

**FIGURE 1:**
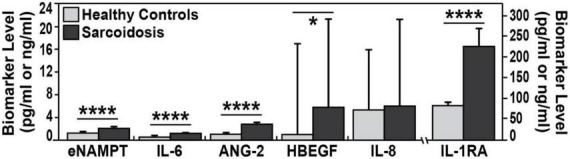
Biomarker plasma levels in sarcoidosis and healthy controls. Plasma levels medians are plotted. Compared to healthy controls, each biomarker exhibited higher values in plasma of sarcoidosis patients. Except for IL-8 all of plasma biomarkers were significantly higher in sarcoidosis. eNAMPT (2.1 vs. 1.2 ng/ml), IL-6 (1.2 vs. 0.6 pg/ml), ANG-2 (2.7 vs. 1.0 pg/ml), HBEGF (5.9 vs. 1 pg/ml), and IL-1 RA (227 vs. 82.3 pg/ml). Mann–Whitney test was calculated for each comparison; ****p-values < 0.0001 and *p < 0.05.

**FIGURE 2:**
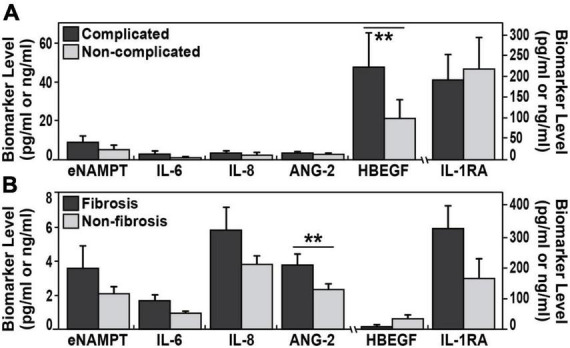
Circulating biomarkers in complicated sarcoidosis and pulmonary fibrosis. **(A)** The plasma levels of HBEGF were significantly elevated (**p < 0.003) in a group of 62 complicated sarcoidosis (parenchymal lung disease by CT scan, radiographic Scadding stages III and IV; Forced vital capacity (FVC) < 50%; cardiac or neurological involvement in addition to lung involvement) compared to non-complicated sarcoidosis. **(B)** ANG-2 plasma levels were significantly (*p* < 0.017) elevated in 19 subjects with sarcoidosis and pulmonary fibrosis (confirmed fibrotic changes on high-resolution chest CT scan or radiographic stage IV) compared to those with non-fibrosis.

Plasma levels of eNAMPT, IL-6, ANG-2, IL-1RA, and HBEGF, but not IL-8 levels, were significantly elevated in sarcoidosis subjects compared to healthy controls (Table 3 and Figure 1). The diagnostic accuracy of the plasma biomarkers was assessed *via* estimates of sensitivity and specificity in addition to 95% confidence intervals and AUCs. IL-1RA exhibited the highest discriminatory accuracy between sarcoidosis and healthy controls (AUC 0.93, 95% CI of 0.90–0.95), followed by ANG-2 (AUC 0.85, 95% CI of 0.79–0.90). eNAMPT, IL-6, and HBEGF exhibited similar AUC values (0.74–0.78) (Figure 2). Spearman correlations to assess inter-marker relationships showed a strong positive correlation between IL-1RA and eNAMPT plasma levels (*r* = 0.42, *p* < 0.00001) and a moderate correlation between IL-1RA and IL-6 levels (*r* = 0.32, *p* < 0.0002). A predictive model with regression analysis was applied to examine the correlation between eNAMPT and IL-1RA and showed IL-1RA values to significantly predict eNAMPT levels (F2,124 = 14.15, prob *F* < 0.001). This significance was not observed for IL-6 levels after adjusting for regression standard error (Supplementary Figure 1).

**TABLE 3:** Biomarker plasma levels in sarcoidosis.

Biomarker	*N*	Median (Q1, Q3)	Sarcoidosis vs. control *P*-value*	Sarcoidosis/pulmonary fibrosis (*n* = 19) *P*-value**	Complicated sarcoidosis (*n* = 62) *P*-value***
eNAMPT (ng/ml)	123	2.1 (1.4,4.3)	**<0.0001**	0.2	0.5153
IL-6 (pg/ml)	123	1.2 (0.8, 1.8)	**<0.0001**	0.18	0.17
IL-8 (pg/ml)	123	3.1 (1.7, 5.3)	0.51	0.7	0.18
IL-1RA (pg/ml)	123	228 (163, 418)	**<0.0001**	0.18	0.35
ANG-2 (ng/ml)	123	2.7 (1.7, 4)	**<0.0001**	**0.017**	0.4
HBEGF (pg/ml)	68	5.9 (1, 39.5)	**0.026**	0.028	**0.003**

Summary statistics and *P*-values from Mann–Whitney test comparing all markers plasma measurements in sarcoidosis vs. healthy controls*; sarcoidosis with pulmonary fibrosis (stage IV) vs. sarcoidosis without pulmonary involvement (stages I and II) ** and complicated sarcoidosis vs. non-complicated sarcoidosis ***. Lung fibrosis is bolded to indicate is an important category, in which the analysis was based.

### Circulating biomarkers in complicated sarcoidosis including pulmonary fibrosis

**FIGURE 3:**
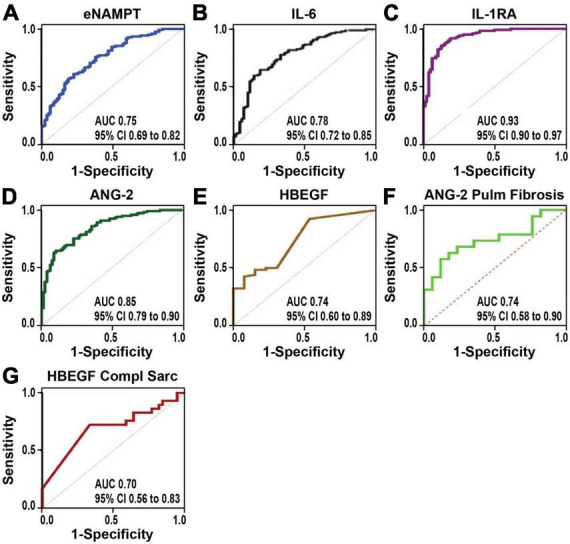
The receiver-operator characteristics curves (ROC) assessing the diagnostic accuracy power. **(A–E)** Sarcoidosis vs. controls. eNAMPT **(A)**, IL-6 **(B)**, IL-1RA **(C)**, ANG-2 **(D)**, and HBEGF **(E)**. IL-1RA showed the highest discriminatory accuracy between sarcoidosis and healthy controls (AUC of 0.93, 95% CI of 0.90–0.95), followed by ANG-2 (AUC 0.85, 95% CI of 0.79–0.90). **(F,G)** Progressive sarcoidosis. ANG-2 diagnostic accuracy performed well to distinguish sarcoidosis pulmonary fibrosis vs. sarcoidosis without pulmonary involvement (Stages I and II) **(F)** and HBEGF showed a good discriminatory power to differentiate complicated sarcoidosis.

We next assessed the capacity for putative sarcoidosis biomarkers to accurately reflect sarcoidosis severity and compared biomarkers levels in 62 subjects with complicated sarcoidosis. In this group, we included subjects with the following; parenchymal lung disease by CT scan, radiographic Scadding stages III and IV; FVC < 50%; cardiac or neurological involvement in addition to lung involvement. This group was compared to biomarkers values in the non-complicated sarcoidosis group (63 subjects). These analyses identified HBEGF as the sole biomarker to significantly differentiate these two groups, with higher mean HBEGF levels in the complicated group, 47.8 pg/ml ± 109.6 vs. 21.4 pg/ml ± 50.5 (AUC 0.70, 95% CI 0.56–0.86, *p* = 0.007) (Figure 2). We next compared biomarker levels in a subgroup of 19 sarcoidosis subjects with pulmonary fibrosis, with confirmed fibrotic changes on high-resolution chest CT scan or radiographic stage IV (Table 1) compared to 17 sarcoidosis subjects without parenchymal lung involvement (Scadding stages II and I) (Figure 2). ANG-2 was the sole marker significantly distinguishing the two groups, (3.8 ± 2.9 ng/ml in fibrosis subjects vs. 2.2 ± 0.8 ng/ml in sarcoidosis subjects without lung involvement, *p* = 0.017), with significant discriminatory power (AUC 0.73, 95% CI 0.57–0.9, *p* = 0.02). We next assessed the ANG-2 cut-off values predictive of pulmonary fibrosis and identified a range of 2.4 ng/ml and 2.8 ng/ml of ANG-2 in plasma to produce a sensitivity of 72 and 66% and a specificity of 65 and 75%, respectively (Figure 3). ANG-2 levels were not significantly different in between sarcoid subjects with pulmonary involvement (Stage III and IV) and subjects without pulmonary involvement (Stage I and Stage II). Summary statistics of all the biomarker levels in controls and sarcoidosis stratified by comparisons groups according to clinical phenotypes are presented in Table 3.

### Sarcoidosis lung and lymph node immunohistochemistry

**FIGURE 4:**
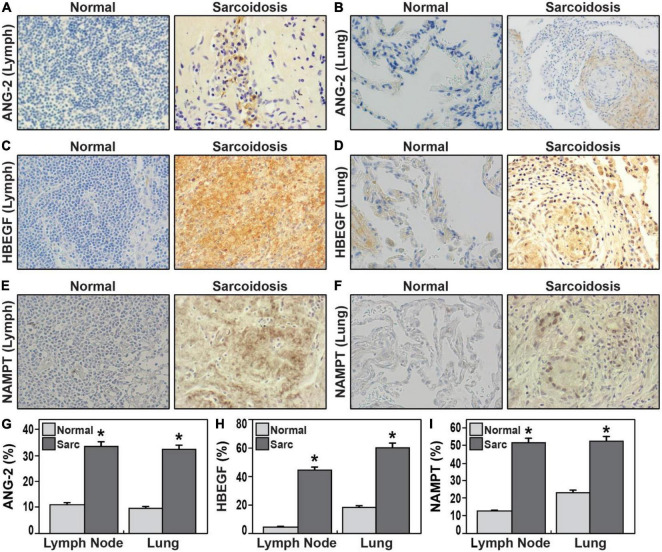
**(A–F)** Representative immunohistochemical staining of lymph nodes **(A,C,E)** and lung tissues **(B,D,F)** from sarcoidosis compared healthy controls 40 × magnification. ANG-2 **(A,B,G)**, HBEGF **(C,D,H)**, NAMPT **(E,F,I)**. **(G–I)** Bar graphs **(G)** illustrate quantification of mean proportions of ANG-2, HBEGF, and NAMPT *p < 0.05. Sarcoidosis in dark gray and normal tissue in light gray. ANG-2 expression in lung tissue and lymph node in sarcoidosis was 32.2 and 33.7%, respectively, in contrast to the expression observed in normal lung and lymph nodes was 9.8 and 11.3%, respectively (*p* = 1.2 e-04). HBEGF expression in sarcoidosis lymph nodes was 44.8% vs. 4.8% in normal lymph nodes (*p* = 3.8 e-05); in normal lungs was 18.3% contrasting with 60.4% in lung of sarcoidosis (*p* = 4.0 e-05). NAMPT mean proportions in lymph nodes were 12.7% in normal tissue and 52% in sarcoidosis (*p* = 4.9 e-04); while in normal lungs was 23.3% vs. 53% in sarcoidosis (*p* = 9.2 e-040. in lymph nodes).

Based upon performance in the immunoassays and association with progression, HBEGF and ANG-2 were next selected for immunohistochemistry staining with Image J quantitation in tissue sections from sarcoidosis lung and lymph node tissue specimens from different donors (Figure 4). We also assessed NAMPT tissue expression due to its strong positive correlation with IL-1RA levels and known linkage to tissue fibrosis (15, 16). HBEGF expression in sarcoidosis lymph nodes was nine times greater than normal lymph node tissues (*p* = 0.000038) and a three-fold increase in expression in sarcoidosis lung compared to normal lung tissues (*p* = 0.00004). Similarly, ANG-2 expression in sarcoidosis lymph node tissues (*p* = 0.0001) and lung tissues (*p* = 0.0001) was significantly higher compared to healthy tissue. Finally, NAMPT expression in sarcoidosis lymph node (*p* = 0.0009) and lung tissues (*p* = 0.005) was significantly higher compared to healthy tissue (Figure 4). These results confirm the elevated expression of the three HBEGF, ANG-2, and eNAMPT plasma biomarkers in sarcoidosis granulomatous tissues.

### Gene expression in sarcoidosis lung tissue

**FIGURE 5:**
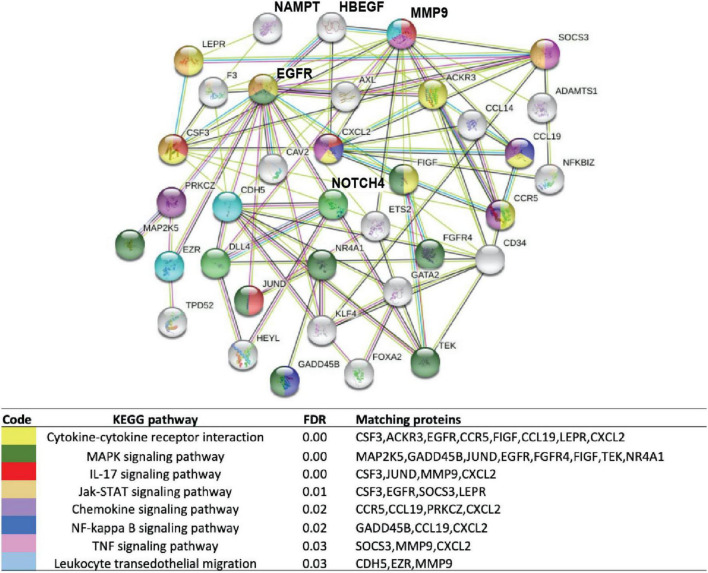
Enrichment analysis of DEGs in lung tissues from subjects with sarcoidosis compared to healthy lung. Functional protein network interaction showing eNAMPT and HBEGF relationship with regulators of lung fibrosis (MMP9), inflammation, and lung remodeling and NOTCH4, a T-cell activity and angiogenesis regulator. Each node represents a coded protein. The edges represent protein–protein associations (line color indicates type of interaction: green—gene neighborhood, red—gene fusion, blue—co-occurrence, and black—co-expression). Pathway analysis and first shell interactors members included are indicated by the color code. A complete list of dysregulated pathways is presented in Supplementary Table 2.

We next interrogated the expression of genes encoding the six plasma biomarkers (*HBEGF, IL-1RA, NAMPT, IL6*, *ANG2*, and *IL8*) using our previously reported publicly available gene expression dataset (GSE157671) generated by NextGen sequencing of micro-dissected sarcoidosis granulomas within lung tissues (6) and mediastinal lymph nodes (11, 32). Comparisons of biomarker panel gene expression failed to identify significant differential expression of the targeted genes between sarcoidosis lymph node granulomas and healthy lymph node tissues. In contrast, *HBEGF* and *NAMPT* were among the 73 DEGs in sarcoidosis lung granulomas (Supplementary Table 2) with significant dysregulation in sarcoidosis lung granulomas compared to lung tissue from controls (FRD 10%, FC1.5). Enrichment analysis of the DEGs and protein–protein interaction analysis using String database (33, 34) demonstrated an association of HBEGF and NAMPT together with proteins involved in inflammatory and lung fibrosis pathways (Figure 5). These included MMP9, a matrix metalloproteinase strongly associated with lung injury and fibrosis (35), and NOTCH4, an important regulator of inflammation and lung remodeling previously implicated in sarcoidosis (4, 32, 36) (Figure 5). A total of 33 KEGG pathways were identified with the top significant dysregulated pathways (*q*-value < 0.03) comprised of cytokine-cytokine receptor interaction, MAPK signaling, IL-17, Jak-STAT signaling, chemokine signaling, NF-kappa B, and TNF signaling pathways (Figure 5). A complete list of all the pathways is presented in Supplementary Table 3. These results are highly consistent with recent studies strongly implicating NAMPT-MMP9 protein interactions in influencing the development and severity of murine radiation-induced lung fibrosis (15, 37).

## Discussion

This study presents a multi-dimensional exploration to validate potential inflammatory and lung remodeling biomarkers with tri-level assessment of circulating plasma levels, tissue protein expression, and lung mRNA transcript expression. With the exception of IL-8, each biomarker in the sarcoidosis panel (eNAMPT, IL-6, ANG-2 IL-1RA, and HBEGF) accurately discriminated between healthy controls and sarcoidosis subjects, with an optimal threshold > 74%. Our plasma eNAMPT results strongly contrast with a previous report indicating no difference between sarcoidosis and healthy controls (38), possibly reflecting differences in studied cohorts as well as biomarker analytic methodologies (commercial ELISA vs. MSD). Each panel biomarker exhibited unique biomarker properties with IL-1RA and ANG-2 exhibiting the most diagnostic accuracy in distinguishing sarcoidosis from healthy subjects. We also identified a strong positive correlation between plasma levels of eNAMPT, IL-1RA, and IL-6 levels, a novel finding in sarcoidosis, previously reported in juvenile idiopathic arthritis (39). The synergistic effect of IL-1 and IL-6 was previously demonstrated to be a necessary factor for T-cell activation in sarcoidosis (22).

ANG-2 and HBEGF proved to be useful biomarkers in assessing disease severity with ANG-2 differentiating sarcoidosis pulmonary fibrosis (stage IV) from sarcoidosis without pulmonary involvement (stages I/II). We noted that when Stage III was incorporated in the analysis, ANG-2 levels were no longer discriminatory, an observation potentially related to the insensitivity of CXRs for assessment of early fibrotic changes of the parenchyma compared to HRCT or PET scans (40). HBEGF plasma levels distinguished a more heterogenous group of complicated sarcoidosis based on the presence of progressive parenchymal lung disease (defined by CT scan, radiographic stages III and IV and/or PFT with FVC < 50%) or vital cardiac or neurological involvement. Peripheral blood concentrations of ANG-2 have been reported to be significantly associated to fibrosis in liver (41) and in idiopathic interstitial pneumonias (23). Furthermore, ANG-2 and HBEGF crosstalk signaling pathways are recognized to upregulate fibronectin synthesis and release of metalloproteinases and collagenases in mesangial cells glomerulosclerosis (16). Our results support this mechanism to be relevant in sarcoidosis pathogenesis as well.

Quantification of biomarker tissue expression by IHC was largely concordant with plasma biomarker results, with elevated expression of NAMPT, HBEGF, and ANG-2 in lung and lymph node tissues from subjects with sarcoidosis. The role of these proteins in the development of sarcoidosis pathobiology is poorly understood. HBEGF signaling responses participate in tissue injury/repair and regeneration, wound healing, and angiogenesis (42). HBEGF serum levels were positively correlated with pulmonary ground-glass score indicative of severe fibrosis (43). Our enrichment analysis of DEGs in lung tissues from subjects with sarcoidosis also revealed the network of interaction of HBEGF and NAMPT with MMP-9, a well-known matrix metalloproteinase target in IPF that is strongly associated with lung injury and fibrosis (35) and associated in progressive sarcoidosis with pulmonary infiltrates or fibrosis (44). MMP9 expression was increased more than 30-fold in sarcoidosis tissues (Supplementary Table 2). These results are highly consistent with our recent studies strongly implicating NAMPT-MMP9 protein interactions in influencing the development and severity of murine radiation-induced lung fibrosis (37). HBEGF interacts with SOCS3, a suppressor of cytokine signaling with an ability to inhibit Jak/STAT signaling, with elevated expression in lymph nodes sarcoidosis (45), we previously identified dysregulated *HBEGF* gene expression in PBMCs sarcoidosis (2). Whether HBEGF is a protective repair response to tissue injury or a direct participant in developing granulomas in sarcoidosis is still unknown, the current study confirms increased HBEGF expression in blood and in lung tissues of sarcoidosis.

Our results also identified other fibrogenesis-associated genes as DEGs in sarcoidosis lungs including *AXL*, involved in migration, aggregation, and anti-inflammation through inhibition of Toll-like receptors (TLRs). *AXL* targeting reduces fibrosis development in experimental renal fibrosis and in human intestinal organoid models (46, 47). Similarly, epidermal growth factor receptor (EGRF) in animal models to study the development of SARS-CoV-induced fibrosis, evidence that pulmonary fibrosis is caused by a hyperactive host response to lung injury mediated by EGFR signaling (48).

The protein interactive network identified an important correlation between HBEGF and NOTCH4, a key regulator of T cell activity and the branching angiogenesis associated with sarcoidosis (4, 32, 36). A GWAS study (4) identified *NOTCH4* SNPs associated with sarcoidosis severity in African Americans. The most dysregulated DEG identified in sarcoidosis tissues was *NPR1*, whose expression was markedly suppressed (> 7 fold reduced expression). Chemokine (C-X-C motif) ligand 2 (CXCL2) also called macrophage inflammatory protein (Figure 5), CXCL2 affects neutrophil recruitment and activation (49) and promotes airway smooth muscle cell migration in asthma induced by IL-17 (50). Expression of chemokine (C–C motif) ligand 19 (CCL19), was markedly increased in sarcoidosis tissues, CCL19 is a critical regulator of the induction of T cell activation. Another highly downregulated gene was NR4A1, a key general regulator in the induction of T cell dysfunction (51). Loss of NR4A1 exacerbates organ fibrosis by dysregulating TGF-β pathway (52, 53) a mechanism potentially related to sarcoidosis lung progressive disease.

The identification of eNAMPT as a sarcoidosis biomarker and candidate gene is highly novel. Secreted eNAMPT, a DAMP and ligand for TLR4 (14), is a therapeutic target (54–56). eNAMPT-triggered inflammation dysregulates signaling pathway that disrupts fibrosis/remodeling resolution. The identification of eNAMPT, as a target and biomarker in sarcoidosis is consistent with the top significant dysregulated pathways identified comprised of cytokine-cytokine receptor interaction, MAPK signaling, IL-17, Jak-STAT signaling, chemokine signaling, NF-kappa B, and TNF signaling pathways (Figure 5). These results also support eNAMPT role in sarcoidosis pathogenesis potentially related to stimulation of CD14 + monocytes, promoting leukocytes and fibroblasts motility through the activation of transcription factors, IL-1RA, TNFα, and IL-6 (22, 25). Together, these data reflect the utility of eNAMPT, ANG-2, and HBEGF as diagnostic blood/tissue markers of sarcoidosis severity and as novel therapeutic targets.

There are limitations to our study including the underpowered nature of the studies performed. Our inability to discern significant IHC differences in marker expression between lung and lymph nodes and differences based on clinical phenotype and the effect of treatment in our results are important limitations. We acknowledge that the biomarkers studied here cannot yet be considered as prognostic biomarkers in newly diagnosed sarcoidosis as the study is not longitudinal and the disease duration was largely unknown in our cohorts. Further investigations are needed to corroborate the gene expression of the targeted markers in an increased number of samples and correlate the plasma/tissue expression and the transcriptome profile with fibrosis progression in sarcoidosis and in other interstitial lung diseases and longitudinal assessment of stability of phenotyping over time.

In summary, the current study has demonstrated the diagnostic accuracy of plasma levels of eNAMPT, IL-6, ANG-2, IL-1RA, and HBEGF as a potential panel of biomarkers for sarcoidosis diagnosis and HBEGF, Enampt, and ANG-2 as markers of sarcoidosis progression and lung fibrosis in subjects with complicated sarcoidosis. Genomic studies corroborated plasma protein dysregulation and identified cytokine-related KEGG pathways in lung granulomatous tissues from sarcoidosis subjects and involvement in inflammatory and lung fibrosis pathways. Together these findings have important implications in the implementation of clinical markers to assess sarcoidosis disease severity and suggest HBEGF, eNAMPT, and ANG-2 may serve as potentially novel indicators of the clinical severity of sarcoidosis disease.

## Data availability statement

The datasets presented in this study can be found in online repositories. The names of the repository/repositories and accession number(s) can be found https://www.ncbi.nlm.nih.gov/geo/query/acc.cgi?acc=GSE157671.

## Ethics statement

The studies involving human participants were reviewed and approved by the University of Arizona (IRB #1509097312R001) and University of Miami (IRB# 20150612). De-identified samples were also obtained from a sarcoidosis cohort from the Instituto Nacional de Ciencias Medicas y Nutrición Salvador Zubirán in Mexico City (Ref. #1711). The patients/participants provided their written informed consent to participate in this study.

## Author contributions

NC, CB, JG, MM, and BS: conception and design of the work, the analysis and interpretation of data for the work, the drafting and revision of the manuscript, and approval of the final version to be published. VR-H, TB, TG, GH-M, JS, BS, SS, JF, and MM: collection and analysis of data, revision of the manuscript, and approval of the final version to be published. VR-H, TB, MH, TG, SS, BS, JF, RO, and SC: data collection and assistance with processing and manuscript production and revision. All authors contributed to the article and approved the submitted version.

## Supplementary material

Supplementary Figure 1 Predictive model with regression analysis was used to assess the relationship between our plasma markers. Scatterplot shows the only significant correlation present, it was between plasma levels of eNAMPT and IL-1RA in sarcoidosis. *Y*-axis shows IL-1RA (pg/mL) and *X*-axis NAMPT (fitted values). Prediction regression model indicates that IL-1RA values significantly predict plasma eNAMPT levels, with an R2 0.19 (F2,124 = 14.15, prob *F* < 0.00).

Supplementary Table 1 The upper limit of detection (ULOD) and lower limit of detection (LLOD) and background concentration of each plasma biomarker.

Supplementary Table 2 The 73 differentially expressed genes in sarcoidosis lung granulomatous tissues compared to healthy lung tissue. Listed by *p*-value.

Supplementary Table 3 KEGG Pathways identified from Differentially Expressed Genes (DEGs) in lung granulomatous tissues from sarcoidosis subjects.
