# Supplementary material for: Biochemical and genomic identification of novel biomarkers in progressive sarcoidosis: HBEGF, eNAMPT, and ANG-2
Source: Front Med (Lausanne). 2022 Oct 25;9:1012827. doi: 10.3389/fmed.2022.1012827 (PMC9640603; doi:10.3389/fmed.2022.1012827)
Supplement: Supplementary file 1 [file Data_Sheet_1.docx]

**Supplementary materials and methods**

**Biomarker plasma measurement**

We utilized 2 different assays to measure the plasma concentration of the biomarker. HBEGF was measured by an ELISA immunoassay, with a detection range 7.8 - 500 pg/ml the background signal was 0.0005, the low limit of detection signal was 0.012, and the lowest concentration in the standard curve was 7.81 pg/ml, as pointed out, the OD was more than twice the lowest concentration of the standard curve. The rest of the biomarkers were assessed using a multiplexed electrochemiluminescent immunoassay more sensitive than the colorimetric ELISA requiring smaller sample volume, and simultaneous processing of multiple biomarkers.

**Immunohistochemistry (IHC) analyses.**

De-identified slides were obtained from the pathology department, samples were obtained from newly diagnosed subjects. All our samples were FFPE, therefore we conducted antigen unmasking (or retrieval) prior to antibody labeling, to avoid the tissue-fixation process that causes cross-linking of proteins. To avoid nonspecific binding of primary and secondary antibodies we added a blocking buffer. Routine H&E slides were prepared using Richard-Allan hematoxylin, clarifier, bluing reagent, and eosin. Then, deparaffinized and rehydrated slides were ringed with an ImmunoPen rinsed in TBS, blocked for endogenous peroxidase (0.5% hydrogen peroxide, 20 min), and protein blocked (Vector Labs, 1 h, avidin D and biotin block, Vector Labs, 250C). To assess alterations in lung and lymph node tissues, the avidin-biotin-peroxidase method was utilized for IHC staining to visualize HB-EGF expression in lung tissues or a Rabbit anti-goat IgG control. Deparaffinized and rehydrated slides were rinsed with an ImmunoPen rinsed in TBS, epitope retrieval citrate at 98° C, 10 min, blocked for endogenous peroxidase using freshly prepared 0.5% hydrogen peroxide, 40 min. Slides were incubated in primary or IgG isotype control, for 15 min at room temperature. After washing, HRP conjugated IgG polymer anti-Rabbit for 8 minutes at room temperature. The protein expression was observed utilizing DAB (Vector Labs), 10 minutes, rinsed in tap water and counterstained with Mayer hematoxylin for 5 min, washed in water, dehydrated, cleared and coverslipped with DPX. Compared to control, marked increases in HB-EGF expression were observed in lung and lymph node tissues with sarcoidosis.

The avidin-biotin-peroxidase method was utilized to visualize NAMPT staining in lung and lymph node tissues. Slides were incubated overnight (4°C) with primary rabbit anti-human NAMPT pAb (Bethyl), β-actin and stained with biotinylated secondary antibody (1 h, 25°C) and imaged (×10 objective, NA 0.4 Zeiss Axiovert photomicroscope; Oberkochen, Germany). a rabbit anti-human NAMPT pAb (1:1000 dilution; Bethyl Laboratories, Montgomery, TX, USA) for immunohistochemistry (IHC) visualization of NAMPT expression in lung tissues. For immunostaining for Ang-2, tissue sections were immersed in 0.3% H2O2 in PBS to block the endogenous peroxidase activity. After rinsing in PBS, the sections were incubated in 10% normal goat serum and then in mouse monoclonal antibody for human Ang-2 (Santa-Cruz Biotechnology; sc-74403) at 4°C overnight. Following a rinse in PBS, they were treated with peroxidase (PO)-conjugated goat anti-mouse IgG for 1h at room temperature. The site of the immunoreaction was visualized by the DAB reaction. Changes in IHC staining were quantitatively summarized by Image J software.
