# Supplementary figures and images for: Biochemical and genomic identification of novel biomarkers in progressive sarcoidosis: HBEGF, eNAMPT, and ANG-2
Source: Front Med (Lausanne). 2022 Oct 25;9:1012827. doi: 10.3389/fmed.2022.1012827 (PMC9640603; doi:10.3389/fmed.2022.1012827)

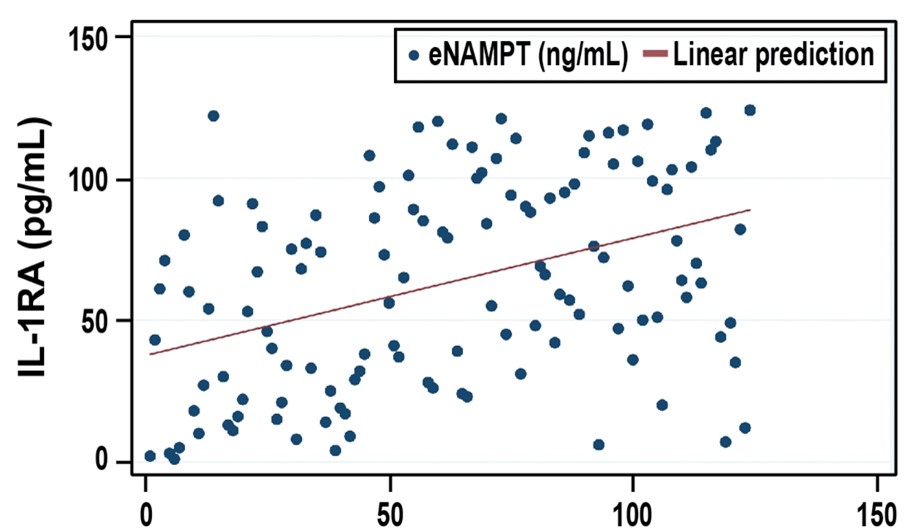

Supplement: Supplementary Figure 1 — Predictive model with regression analysis was used to assess the relationship between our plasma markers. Scatterplot shows the only significant correlation present, it was between plasma levels of eNAMPT and IL-1RA in sarcoidosis. Y-axis shows IL-1RA (pg/mL) and X-axis NAMPT (fitted values). Prediction regression model indicates that IL-1RA values significantly predict plasma eNAMPT levels, with an R2 0.19 (F2,124 = 14.15, prob F < 0.00). [file Image_1.JPEG]
